# Genome-wide mapping of sister chromatid exchange events in single yeast cells using Strand-seq

**DOI:** 10.7554/eLife.30560

**Published:** 2017-12-12

**Authors:** Clémence Claussin, David Porubský, Diana CJ Spierings, Nancy Halsema, Stefan Rentas, Victor Guryev, Peter M Lansdorp, Michael Chang

**Affiliations:** 1 European Research Institute for the Biology of Ageing, University Medical Center GroningenUniversity of GroningenGroningenNetherlands; 2 Terry Fox LaboratoryBC Cancer AgencyVancouverCanada; 3 Department of Medical GeneticsUniversity of British ColumbiaVancouverCanada; Columbia UniversityUnited States

**Keywords:** sister chromatid exchange, double-strand break, homologous recombination, DNA repair, Strand-seq, single cell sequencing, *S. cerevisiae*

## Abstract

Homologous recombination involving sister chromatids is the most accurate, and thus most frequently used, form of recombination-mediated DNA repair. Despite its importance, sister chromatid recombination is not easily studied because it does not result in a change in DNA sequence, making recombination between sister chromatids difficult to detect. We have previously developed a novel DNA template strand sequencing technique, called Strand-seq, that can be used to map sister chromatid exchange (SCE) events genome-wide in single cells. An increase in the rate of SCE is an indicator of elevated recombination activity and of genome instability, which is a hallmark of cancer. In this study, we have adapted Strand-seq to detect SCE in the yeast *Saccharomyces cerevisiae*. We provide the first quantifiable evidence that most spontaneous SCE events in wild-type cells are not due to the repair of DNA double-strand breaks.

## Introduction

Homologous recombination (HR) is indispensable for the maintenance of genome integrity, and mutations in many HR genes are linked to a number of human diseases, especially various types of cancer (Prakash et al., 2015). HR is characterized as a major pathway to repair DNA double-strand breaks (DSBs), and can occur between any two homologous sequences, such as sister chromatids, homologous chromosomes, or homologous sequences located at different genomic loci (Jasin and Rothstein, 2013). HR results in a unidirectional transfer of genetic information from one DNA molecule to another (i.e. gene conversion) and/or a reciprocal exchange of genetic information between the two DNA molecules (i.e. a crossover). A crossover involving two homologous chromosomes can cause loss of heterozygosity, which is a common occurrence in cancer, while a crossover involving homologous sequences located at different genomic loci will result in chromosomal rearrangements, such as deletions, inversions, and translocations, which have been linked to a variety of human diseases (Kong et al., 2011). Thus, in mitotic cells, HR is biased towards noncrossovers, and HR involving identical sister chromatids, referred to as sister chromatid recombination (SCR), is preferred (Kadyk and Hartwell, 1992; Nassif et al., 1994; Johnson and Jasin, 2000; Virgin et al., 2001; Stark and Jasin, 2003), in part because a crossover during SCR should not lead to a change in DNA sequence.

However, studying SCR has been difficult, precisely because it does not normally result in a change in DNA sequence. Nevertheless, an SCR event that leads to a crossover, referred to as a sister chromatid exchange (SCE), can be detected. The mechanistic details of SCE and HR have been best studied using genetic assays in the budding yeast *Saccharomyces cerevisiae* (Symington et al., 2014), though these assays are limited in several ways. First, perfect, or ‘equal’, chromosomal SCE events cannot be detected genetically, so most SCE assays measure ‘unequal’ SCE events, requiring recombination between misaligned sister chromatids. However, using a plasmid-based assay that creates a DSB on one of the two sister plasmids after DNA replication, equal SCE was found to occur much more frequently than unequal SCE (González-Barrera et al., 2003). Second, an often-used approach for detecting unequal SCE involves using two overlapping segments of a reporter gene in a tail-to-head configuration (Fasullo and Davis, 1987). The generation of a full-length reporter gene can result from an unequal SCE event, but it can also result from a long-tract gene conversion event, which does not involve a crossover. These two outcomes can be distinguished by analysis of sectored colonies (Kadyk and Hartwell, 1992), but this additional analysis is often not performed. Third, these assays measure recombination at a single genetic locus by introducing genetic markers at the locus. Introduction of such ‘reporter cassettes’ may disrupt the native chromatin environment, which could affect HR at the locus. Assaying recombination only at a single locus also ignores the rest of the genome. Some proteins important for HR may only affect specific regions of the genome, so these assays would not be useful to elucidate their functions. Lastly, most genetic assays rely on the outgrowth of a recombined cell. Thus, recombination events that lead to a reduction in cell proliferation capacity will be underrepresented, or left uncharacterized.

**Figure 1.**
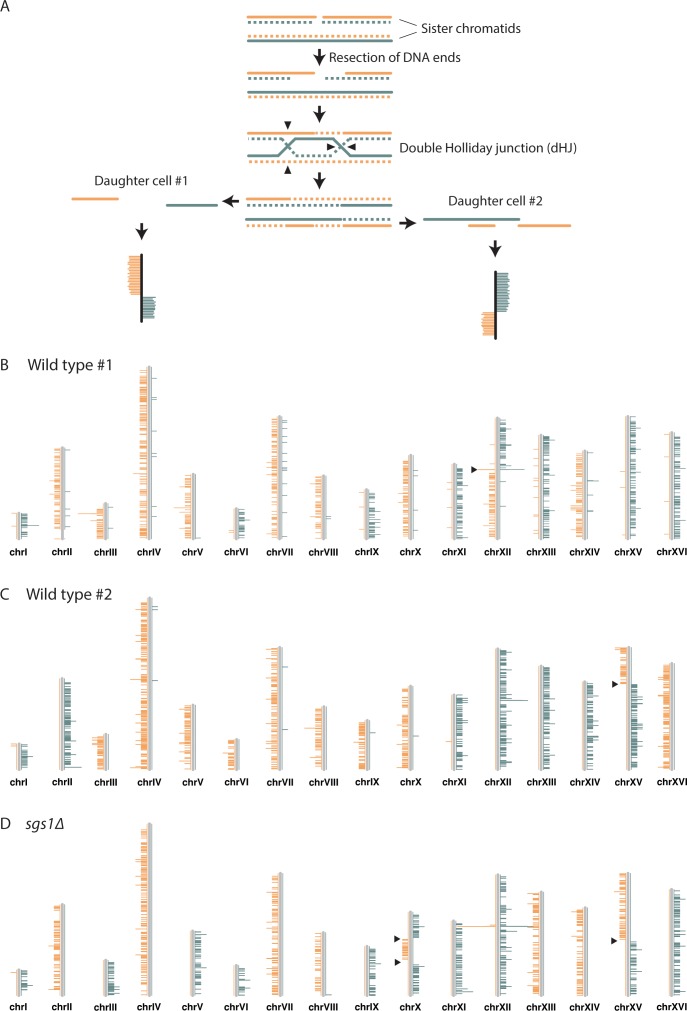
Detection of SCE events using Strand-seq. (**A**) An SCE can occur as a result of DSB repair. Two sister chromatids, one of which has a DSB, are shown. The parental template DNA strands are depicted with solid lines, while the newly synthesized strands containing BrdU are depicted with dashed lines. The Watson and Crick strands are shown in orange and blue, respectively. DSB repair by SCR can lead to the formation of a double Holliday junction (dHJ). Resolution of the dHJ by structure-specific endonucleases will result in either a noncrossover (not shown) or a crossover. The resulting sister chromatids are then segregated to two different daughter cells. In the current Strand-seq protocol, only one daughter cell is isolated and analyzed. The BrdU-containing strands are nicked during library preparation, resulting in the sequencing of only parental strands. Sequence reads are mapped to either side of a chromosome ideogram. An SCE results in a switch from Watson to Crick reads along the chromosome. Note: the small gap between the parental strands in daughter cell #1 and the small overlap of the parental strands in daughter cell #2 are too small to be detected with Strand-seq. (**B**) An example of a wild-type Strand-seq library. Ideograms of the 16 yeast chromosomes are shown. Orange and blue lines correspond to reads aligning to the Watson and Crick strands, respectively. This cell inherited either the parental Watson strand or the parental Crick strand for each chromosome, except chromosome XII. A switch from Watson to Crick reads can be seen for chromosome XII (black arrowhead), indicating that an SCE event has occurred. (**C**) A second example of a wild-type Strand-seq library. An SCE event was detected on chromosome XV. (**D**) An example of an *sgs1∆* Strand-seq library. Three SCE events were detected in this library: two on chromosome X and one on chromosome XV.

To alleviate these limitations, we previously developed a DNA template strand sequencing technique, called Strand-seq, which is capable of detecting SCE events genome-wide in single cells (Falconer et al., 2012). In Strand-seq, cells are allowed to divide once in the presence of bromodeoxyuridine (BrdU), a synthetic analogue of thymidine. BrdU gets incorporated into the newly synthesized DNA strands. Single daughter cells are isolated and the nascent DNA strands are selectively degraded to isolate parental template strands for the construction of directional sequencing libraries. Multiple single-cell libraries containing unique index sequences are pooled and sequenced on an Illumina platform. Aligned reads are binned into non-overlapping segments and plotted as coloured horizontal lines along an ideogram of each chromosome. An SCE is revealed by a switch in reads mapping to the Watson strand to reads mapping to the Crick strand, or vice versa (Figure 1A). Strand-seq was originally developed for use with mammalian cells. However, since most mammalian cells are diploid, it is not easy to differentiate between an SCE from an interhomolog recombination event. Yeast, on the other hand, can be studied in both haploid and diploid states, and the molecular details of HR are best known from studies in yeast. Thus, we have adapted the Strand-seq approach to work with yeast cells. We observe that 23% of wild-type cells have at least one SCE—the first time spontaneous SCEs have been measured and mapped genome-wide in yeast. Interestingly, our findings suggest that most spontaneous SCE events are not due to the repair of DSBs.

**Figure 1—figure supplement 1.**
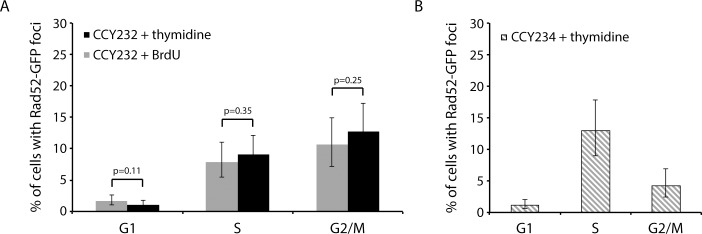
Incorporation of BrdU does not increase Rad52 focus formation during the first cell cycle. (**A**) The percentages of G1, S, and G2/M cells with at least one Rad52-GFP focus are shown for CCY232 (isogenic to the E17 wild-type Strand-seq strain, except with *RAD52-GFP*) grown in the presence of thymidine or BrdU for 90 min (thymidine: G1 = 18/1058, S = 32/404.5, G2/M = 25.5/240.5; BrdU: G1 = 10.5/1009, S = 37.5/415, G2/M = 35/275). Numbers are averages of cells counted by two people independently. (**B**) As an additional control, the percentages of G1, S, and G2/M cells with at least one Rad52-GFP focus were determined for CCY234 (a wild-type ‘non-Strand-seq’ strain that expresses Rad52-GFP; G1 = 9.5/823, S = 31/240, G2/M = 13.5/318.5), and are similar to previously published data (Lisby et al., 2001). Error bars show 95% confidence intervals. P-values were calculated using Fisher’s exact test.

## Results

### Analyzing sister chromatid exchange in single yeast cells using Strand-seq

**Figure 2.**
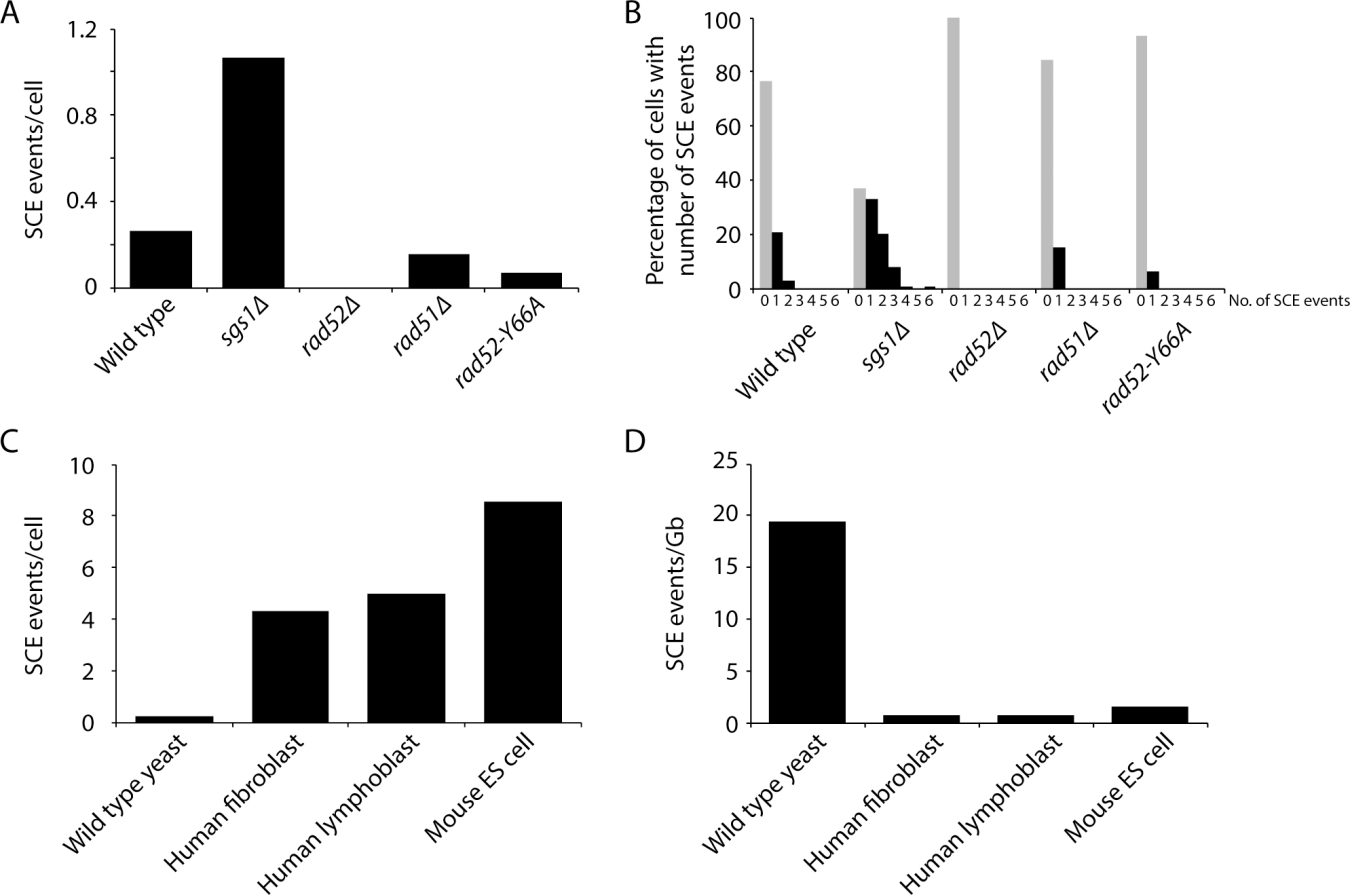
Measurement of spontaneous SCE genome-wide in single cells. (**A**) Number of SCE events per cell for the indicated genotypes. (**B**) Percentage of cells with the indicated number of SCE events for each genotype. (**C**) Number of SCE events per cell for wild-type yeast, human fibroblasts, human lymphoblasts, and mouse ES cells. (**D**) SCE events per gigabase of DNA for wild-type yeast, human fibroblasts, human lymphoblasts, and mouse ES cells.

*S. cerevisiae* cells are unable to ‘salvage’ nucleosides from the environment, so to perform Strand-seq with yeast cells, we use yeast strains that express both *Drosophila melanogaster* deoxyribonucleoside kinase (dNK) and the human equilibrative nucleoside transporter (hENT1), allowing cells to take up and incorporate exogenous thymidine and BrdU into their DNA (Vernis, 2003). *CDC21*, which encodes thymidylate kinase, is also deleted in these strains, forcing them to be entirely dependent upon exogenous thymidine for viability. When grown in the presence of BrdU, these strains do not show any cell cycle delay or DNA damage checkpoint activation during the first cell cycle (Vernis, 2003). In addition, we see no significant difference in Rad52 focus formation, a sensitive indicator of recombination activity (Lisby et al., 2001), after 90 min of growth in BrdU-containing medium, suggesting that BrdU does not stimulate SCE (Figure 1—figure supplement 1). Although we cannot exclude the possibility that BrdU incorporation causes DNA damage that induces SCE in a manner that does not elicit a DNA damage checkpoint response or Rad52 focus formation, these findings are in line with our recently reported observation that BrdU incorporation also does not induce SCE in human cells, at least during the first two cell divisions, as varying the concentration of BrdU in the culture medium does not affect the frequency of SCE (van Wietmarschen and Lansdorp, 2016). We release dNK- and hENT1-expressing *cdc21∆* yeast cells synchronously from a G1 arrest into medium supplemented with BrdU and monitor cell cycle progression by flow cytometry. Cells that undergo precisely one cell division are isolated by cell sorting, followed by single cell Strand-seq library preparation (Sanders et al., 2017). Two examples of wild-type Strand-seq libraries, each containing one SCE, are shown in Figure 1B and C. We sequenced Strand-seq libraries derived from 218 wild-type cells and observed a total of 57 SCE events, which equates to 0.26 SCE events per cell (Figure 2A, Table 1). 23% of wild-type cells have at least one SCE (Figure 2B).

**Table 1.** Comparison of SCE by genotype.

Genotype	No. of cells analyzed	No. of SCEs (SCEs/cell)	No. of SCEs at rDNA	SCEs/Gb (outside rDNA)	SCEs/Gb (within rDNA)
Wild type	218	57 (0.26)	14	16.3	47.0
*sgs1∆*	103	110 (1.07)	25	68.2	177.8
*rad52∆*	27	0 (0)	0	0	0
*rad51∆*	65	10 (0.15)	6	5.1	67.6
*rad52-Y66A*	76	5 (0.07)	4	1.1	38.6

To validate our approach, we analyzed SCE in a hyperrecombinogenic strain (*sgs1∆*), and in a strain lacking Rad52 (a key HR protein important for all HR-mediated activities). Mutation of BLM, the human homolog of *SGS1*, increases SCE (German et al., 1965; Chaganti et al., 1974; van Wietmarschen and Lansdorp, 2016), and deletion of *SGS1* has been reported to increase unequal SCE using two different genetic assays (Onoda et al., 2000; Fasullo and Sun, 2017). The evolutionarily conserved Sgs1-Top3-Rmi1 complex is thought to suppress SCE by dissolving a recombination intermediate known as the double Holliday junction (dHJ; see Figure 1A) in a manner that would result in a non-crossover (Ira et al., 2003; Wu and Hickson, 2003). Consistent with these findings, we observe an increase in SCE in *sgs1∆* cells (p<10^−9^) using Strand-seq (Figure 1D; Figure 2A and B; Table 1). In addition, we did not detect any SCE events in *rad52∆* cells, as expected (Figure 2A and B; Table 1).

The Strand-seq approach allows us to directly and quantitatively compare recombination activity in cells from yeast and mammals. Using previously reported Strand-seq data from mouse embryonic stem cells (Falconer et al., 2012) and human fibroblasts and lymphoblasts (van Wietmarschen and Lansdorp, 2016), we find that spontaneous SCE events per cell is about an order of magnitude greater in human and mouse cells than in wild-type yeast cells (Figure 2C). However, the human and mouse genomes are much larger than the yeast genome. By plotting the number of SCE events per gigabase of DNA, it is apparent that SCE activity is about 20-fold higher in yeast (Figure 2D). It is generally thought that yeast is more recombinogenic than mammals, but a direct and quantitative comparison has not been available until now.

### Sister chromatid exchange at the ribosomal DNA locus

**Figure 3.**
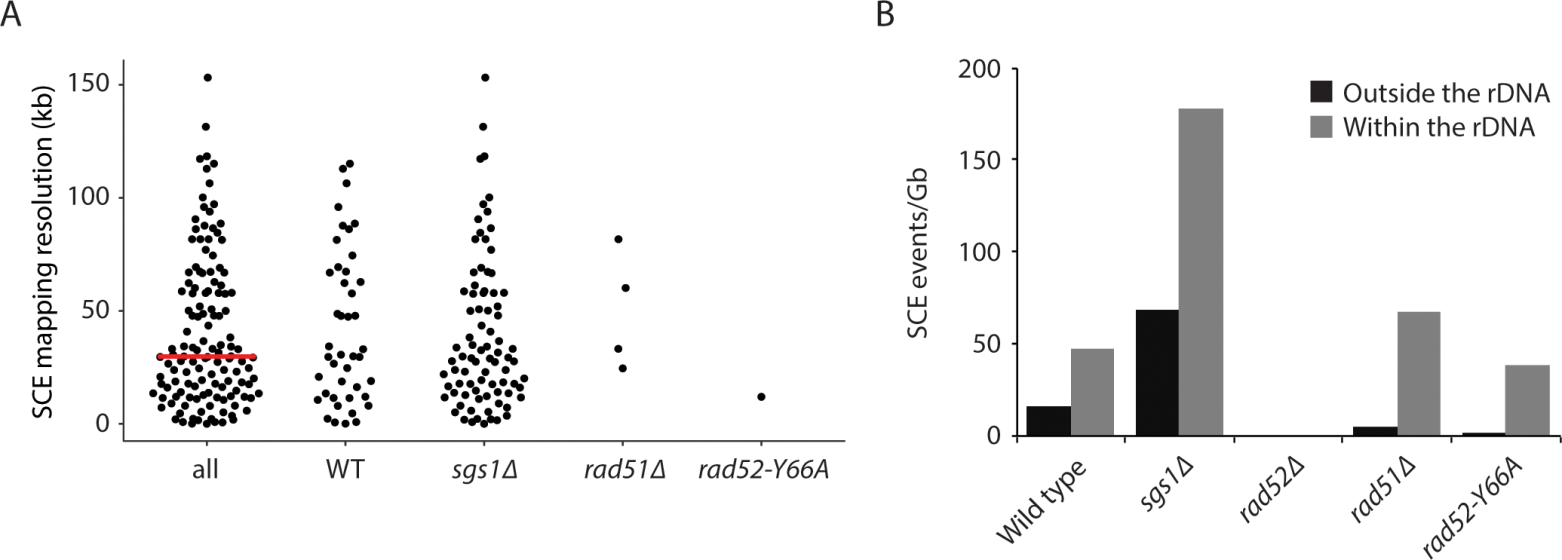
Mapping SCE events. (**A**) Mapping resolution of SCE events in all yeast Strand-seq libraries and by genotype. The red line shows the median mapping resolution for all libraries. SCE events within the rDNA locus are excluded from this analysis because it is not possible to determine where within the rDNA an SCE event has occurred. (**B**) SCE events per gigabase of DNA, for either the entire genome excluding the rDNA locus or only considering the rDNA locus, were plotted for the indicated genotypes.

Currently, we are only able to map SCE events to a resolution of approximately 30 kb (Figure 3A), which, combined with the modest number of SCE events we have detected so far (57 total from 218 wild-type Strand-seq libraries; Table 1), prevents us from mapping SCE events to specific genomic loci or features. However, multiple SCE events were seen at the ribosomal DNA (rDNA) locus, which is located on chromosome XII and consists of approximately 150 rDNA repeats, each of which is 9.1 kb in size (the SCE event in the Strand-seq library shown in Figure 1B maps to the rDNA locus). Since the repeats are identical in sequence, we cannot determine where within the ~1.4 Mb rDNA locus an SCE event occurs. Therefore, we only report whether or not an SCE has occurred somewhere within the rDNA locus. Of the 57 spontaneous SCE events we observed in our wild-type Strand-seq libraries, 14 were found at the rDNA locus (Table 1). We find that the rate of spontaneous SCE per gigabase of DNA in the rDNA locus is about threefold higher compared to the rest of the genome. This result was observed both in the presence and absence of Sgs1 (Figure 3B), and is likely due to the presence of the replication fork barrier within each rDNA repeat (Labib and Hodgson, 2007).

### The strand annealing activity of Rad52 is important for sister chromatid exchange

Rad52 has two main biochemical functions. First, Rad52 mediates the exchange of replication protein A (RPA) for Rad51 on single-stranded DNA (ssDNA) to promote Rad51-catalyzed strand invasion (Sung, 1997; New et al., 1998). Second, Rad52 facilitates the annealing of complementary RPA-coated ssDNA, a function that is independent of Rad51 (Mortensen et al., 1996; Shinohara et al., 1998; Sugiyama et al., 1998) and augmented by Rad59 (Petukhova et al., 1999; Davis and Symington, 2001; Wu et al., 2006). To determine which function of Rad52 is important for SCE, we performed Strand-seq on *rad51∆* and *rad52-Y66A* mutant cells. *rad52-Y66A* belongs to a class of *rad52* mutants, class C mutants, that are mutated in the evolutionarily conserved N-terminal domain and share a similar phenotype of being proficient for mitotic recombination, but defective in the repair of DSBs (Mortensen et al., 2002). An in vitro study of one of the class C mutant proteins, rad52-R70A, indicated that these mutants are defective in strand annealing activity (Shi et al., 2009). We find that SCE is reduced 1.7-fold in *rad51∆* cells and 4.5-fold in *rad52-Y66A* cells (p=0.058 and p<10^−4^ for *rad51∆* and *rad52-Y66A*, respectively; Figure 2A and B; Table 1), indicating that both functions of Rad52 are important for spontaneous SCE, with the strand annealing function being more so. This observation is in agreement with previous work indicating that the strand annealing activity of Rad52 is important for DSB-induced SCR (Muñoz-Galván et al., 2013). Using a genetic assay that detects unequal SCE, it was previously reported that Rad51 is not required for spontaneous SCE (Fasullo et al., 2001). However, this study did not assess the contribution of long-tract gene conversion in their assay, which may have obscured an actual decrease in unequal SCE in the *rad51∆* mutant.

Interestingly, the effect of *rad51∆* and *rad52-Y66A* on SCE is restricted to outside of the rDNA locus (3.2-fold [p=0.002] and 15-fold [p<10^−6^] reduction, respectively, compared to wild type; Figure 3B). Within the rDNA locus, SCE in *rad51∆* and *rad52-Y66A* is not significantly altered (p=0.48 and p=0.71, respectively). This observation is consistent with a previous study showing that spontaneous Holliday junction formation within the rDNA locus is dependent on Rad52, but not Rad51 (Zou and Rothstein, 1997). Taken together, the evidence suggests that Rad52 may have a function independent of Rad51 and strand annealing at the rDNA locus.

### Double-strand breaks are not the main cause of spontaneous sister chromatid exchange

It is often thought that SCE events are the result of the repair of DSBs (as depicted in Figure 1A). To investigate the role of DSBs in the generation of spontaneous SCE events in yeast, we compared the rate of spontaneous SCE in wild-type cells to the rate of DSB formation. Spontaneous DSB rate can be estimated by the rate of cell death in a *rad52∆* mutant, which cannot repair DSBs (Coïc et al., 2008; Mehta and Haber, 2014). A single unrepaired DSB is sufficient to kill a *rad52* mutant cell (Weiffenbach and Haber, 1981). Thus, we used the mortality rate of our *rad52∆* strain to estimate the rate of spontaneous DSB formation, which we found to be 12% per cell division (see Materials and methods). Using direct-repeat recombination assays to detect unequal SCR events, one quarter of spontaneous (two of eight recombinants) and one third of X-ray-induced (four of 12 recombinants) SCR events were found to be accompanied by a crossover (Jackson and Fink, 1981; Kadyk and Hartwell, 1992). Therefore, if all spontaneous SCE events are the result of DSB repair, the rate of spontaneous SCE should be 3–4%. This rate represents an upper limit, because if Rad52 repairs other types of DNA damage that could lead to cell death if unrepaired, we would have overestimated the rate of spontaneous DSB formation. In contrast to this 3–4% upper limit, we find that 20% (43 out of 218, 95% CI [15%, 26%]) of wild-type Strand-seq libraries have at least one SCE not at the rDNA locus. We exclude SCE events that map to the rDNA locus because a DSB in the rDNA locus can be repaired by single-strand annealing in the absence of Rad52 due to the highly repetitive nature of the locus (Ozenberger and Roeder, 1991). Therefore, our results suggest that the majority of spontaneous SCE events are not the result of DSB repair.

**Figure 4.**
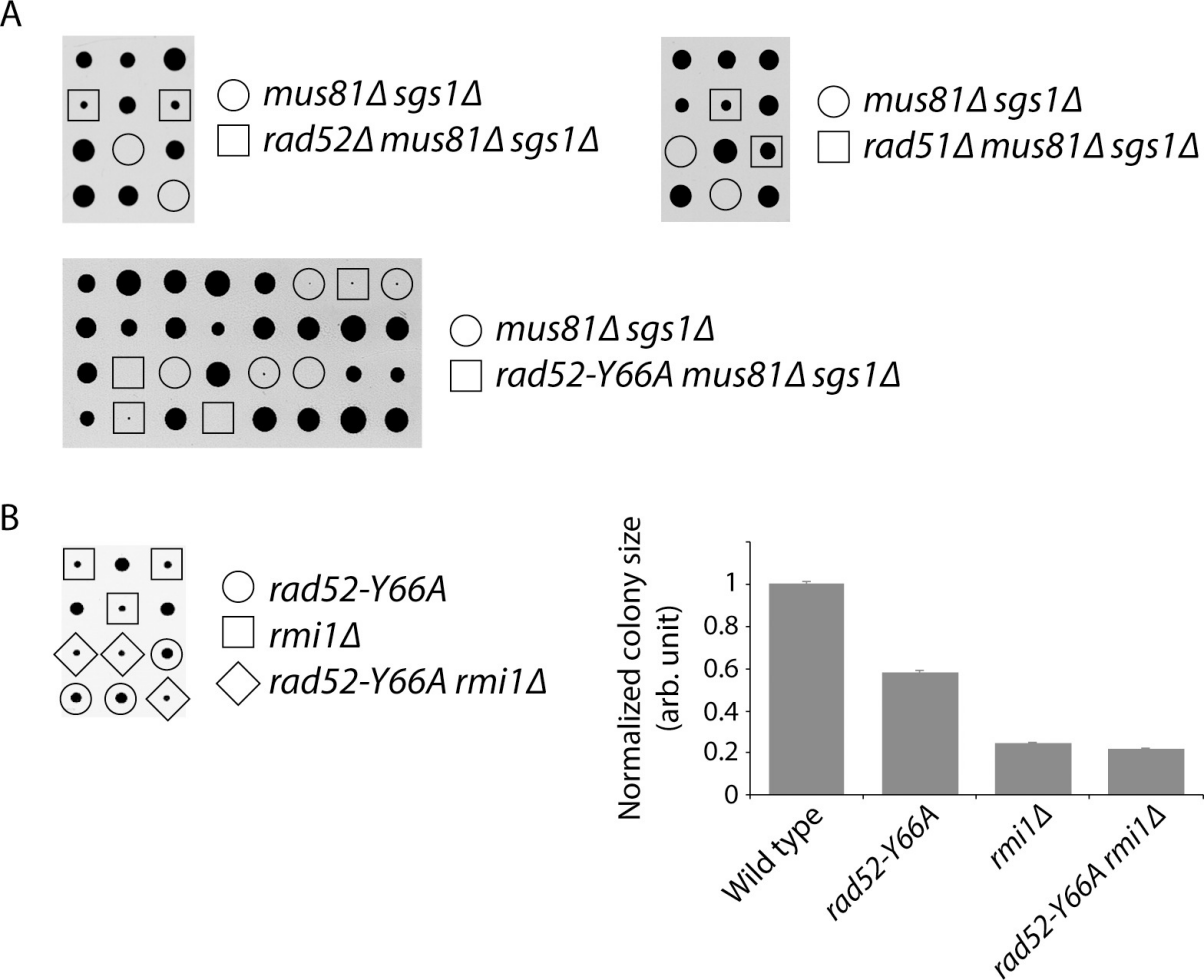
Abolishing the strand annealing activity of Rad52 does not suppress *mus81∆ sgs1∆* synthetic lethality or *rmi1∆* slow growth. (**A**) Representative tetrads derived from the sporulation of MCY736, MCY737, and MCY773 are shown. (**B**) Representative tetrads derived from the sporulation of CCY198 are shown. Colony sizes for the indicated genotypes were measured and normalized to wild type. Mean ±SEM is shown. Lack of suppression of *mus81∆ sgs1∆* synthetic lethality and *rmi1∆* slow growth was also observed using another *rad52* class C mutant: *rad52-R70A* (data not shown).

Spontaneous HR initiated by non-DSB damage has been reported before, so we investigated whether the same lesion could be responsible for spontaneous SCE. For example, cells lacking both Sgs1 and the structure-specific endonuclease Mus81 are inviable, but this lethality can be suppressed by mutations in the *RAD52* epistasis group (i.e. *RAD51*, *RAD52*, *RAD54*, *RAD55*, and *RAD57*), indicating that *mus81∆ sgs1∆* synthetic lethality is due to a toxic recombination intermediate (Fabre et al., 2002). Since Rad52 is required for the repair of DSBs, Fabre and colleagues argued that the toxic recombination intermediate cannot be initiated by a DSB. Similarly, cells lacking Top3 or Rmi1 grow very poorly, but this slow growth can be suppressed by mutations in *SGS1* or the members of the *RAD52* epistasis group, supporting a model where Rad52 and Sgs1 function in a pathway to repair a spontaneous non-DSB lesion, in the process creating a toxic recombination intermediate requiring Top3 and Rmi1 to resolve (Gangloff et al., 1994; Shor et al., 2002; Chang et al., 2005; Mullen et al., 2005). Unlike *rad51∆* and *rad52∆*, which can suppress the synthetic lethality of *mus81∆ sgs1∆* and the slow growth of *rmi1∆* (Fabre et al., 2002; Chang et al., 2005), we find that *rad52* class C mutants, which are defective in Rad52-mediated strand annealing (Shi et al., 2009) and spontaneous SCE (Figure 2A and B), cannot suppress either phenotype (Figure 4). These findings indicate that the DNA lesion(s) that cause *mus81∆ sgs1∆* synthetic lethality and *rmi1∆* slow growth are different than the DNA lesion(s) that cause spontaneous SCE in wild-type cells, and that HR is important to repair multiple types of endogenous DNA damage.

## Discussion

**Figure 5.**
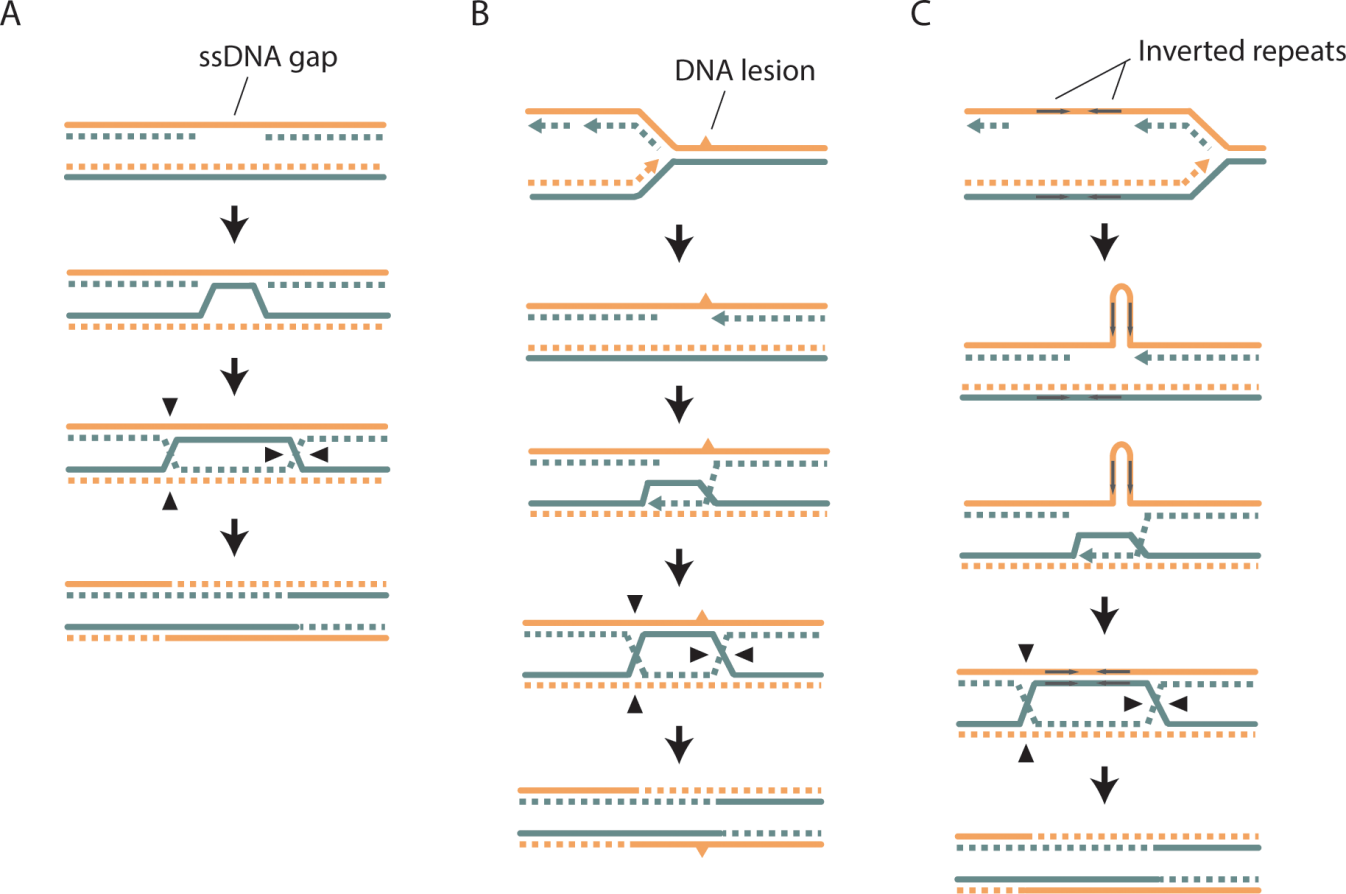
Models of SCE that do not involve a DSB. As in Figure 1A, the parental template DNA strands are depicted with solid lines, while the newly synthesized strands are depicted with dashed lines. The Watson and Crick strands are shown in orange and blue, respectively. (**A**) An SCE could be generated from the repair of an ssDNA gap. This could proceed via inverse strand exchange, where Rad52 forms a complex with dsDNA and promotes strand exchange with a homologous ssDNA sequence independently of Rad51 (Mazina et al., 2017). (**B**) A DNA lesion on one of the parental template strands can cause template switching, where nascent DNA is used as a template for DNA replication, and could result in SCE. (**C**) As in **B**), but the ‘DNA lesion’ is an inverted DNA repeat forming a hairpin loop.

In this study, we have used Strand-seq to measure SCE genome-wide in individual yeast cells. We provide evidence that the majority of spontaneous SCE events in wild-type cells are not the result of DSB repair. Non-DSB lesions have also been invoked to explain the synthetic lethality of *mus81∆ sgs1∆* and *sgs1∆ srs2∆* mutants (Fabre et al., 2002) and the proficiency of *rad52* class C mutants for spontaneous inter- and intrachromosomal heteroallelic HR (Lettier et al., 2006). While the strand annealing activity of Rad52 is important for spontaneous SCE, it is not in the other noted instances of spontaneous HR, indicating that there must be at least two types of endogenous non-DSB lesions processed by HR. These lesions could be single-stranded nicks or gaps, as proposed in many of the original models for HR (Holliday, 1964; Meselson and Radding, 1975; Radding, 1982), or other type of structures interfering with strand-specific replication (Figure 5A and B). Indeed, it is well known that single-stranded gaps can initiate recombination in *Escherichia coli* (Persky and Lovett, 2008) and that fork collapse at the polar replication fork barrier *RTS1* in *Schizosaccharomyces pombe* induces HR-dependent replication restart from a single-stranded gap (Lambert et al., 2010).

The importance of strand annealing for spontaneous SCE is reminiscent of recombination involving inverted repeats, which requires Rad52 but is only modestly reduced by deletion of *RAD51* (Rattray and Symington, 1994). Rad59, which augments the strand annealing function of Rad52 (Petukhova et al., 1999; Davis and Symington, 2001; Wu et al., 2006), is required for spontaneous recombination of inverted repeats in the absence of Rad51 (Bai and Symington, 1996). It has been proposed that these events occur by template switching during DNA replication in a manner involving strand annealing (Mott and Symington, 2011). Poly-ubiquitylation of PCNA by the Mms2-Ubc13-Rad5 ubiquitin conjugating enzyme complex promotes repair through the error-free post-replication repair pathway, which is thought to involve template switching (Xu et al., 2015). Rad5 and Rad18 were found to have only a minor role in inverted-repeat recombination (Mott and Symington, 2011), and the rates of spontaneous SCE in *mms2∆*, *ubc13∆*, and *rad5∆* mutants are similar to wild type, as measured by an unequal SCE assay (Fasullo and Sun, 2017). However, template switching has been proposed to occur differently depending on whether the DNA lesion is on the leading strand template or the lagging strand template, with lesion bypass on the leading strand utilizing Rad5 while lesion bypass on the lagging strand mediated by Rad52 (Gangavarapu et al., 2007). Aside from the rDNA locus, we have so far been unable to map spontaneous SCE events to specific genomic features so further work will be needed to determine whether inverted repeats replicated by the lagging strand machinery are a source of spontaneous SCE (Figure 5C).

We were able to map multiple spontaneous SCE events to the rDNA locus because of its large size (approximately 10% of the genome). Due to its repetitive nature, the rDNA locus has been an excellent substrate for studying HR. Recombination within the locus is important to maintain repeat homogeneity and copy number homeostasis (Kobayashi, 2011). Consistent with the importance of HR at the rDNA locus, we observe that SCE is threefold higher at the rDNA locus compared to the rest of the genome (Figure 3B). Nevertheless, an SCE event occurs at the rDNA locus in only 6.4% (14 out of 218) of wild-type cells (Table 1). Considering that it has been estimated that a single cell has on average 3.6 Holliday junctions and one DSB within the rDNA array per cell cycle (Zou and Rothstein, 1997; Sasaki and Kobayashi, 2017), our data indicate that the vast majority of HR events at the rDNA locus do not result in crossovers, in agreement with previous work showing that HR at the rDNA locus is largely rearrangement-free (Zou and Rothstein, 1997).

In addition to SCE, Strand-seq could in theory be used to detect break-induced replication (BIR) events involving sister chromatids. BIR occurs when one end of a DSB invades an intact homologous DNA sequence to generate a replication fork that can potentially proceed to the end of the chromosome. Unlike canonical DNA replication, which is semi-conservative, BIR occurs by conservative DNA synthesis (Donnianni and Symington, 2013; Saini et al., 2013). With Strand-seq, a chromosomal region synthesized by BIR could either have no Watson or Crick reads (i.e. both Watson and Crick strands are newly synthesized) or both Watson and Crick reads (i.e. both Watson and Crick strands consist of the original parental template strands). Application of Strand-seq in yeast will be particularly advantageous to address questions about various types of DNA lesions and replication stress-inducing barriers at specific locations in the genome. These and other potential applications of Strand-seq offer intriguing new possibilities to study HR, complimenting more traditional assays to yield a better picture of HR activity genome-wide at the level of single cells.

## Materials and methods

### Yeast strains and growth conditions

Strains used in this study are listed in Table 2 and are all *RAD5* derivatives of W303 (Thomas and Rothstein, 1989; Zhao et al., 1998). The E17 strain was derived from YLV11 (Vernis, 2003). Unless noted otherwise, standard culturing conditions and genetic techniques were used to manipulate yeast strains (Sherman, 2002).

**Table 2.** Yeast strains used in this study.

Strain name	Relevant genotype	Source
E17	*MAT***a** *ADE2 cdc21::kanMX leu2::LEU2-GAL-hENT1 LYS2 RAD5 trp1::TRP1-GAL-dNK ura3-1*	Peter Thorpe
CCY232	*MAT***a** *ADE2 cdc21::kanMX leu2::LEU2-GAL-hENT1 LYS2 RAD5 trp1::TRP1-GAL-dNK ura3-1 RAD52-GFP::HIS3M × 6*	This study
CCY234	*MAT***a** *ADE2 can1-100 his3-11,15 leu2-3,112 trp1-1 ura3-1 RAD5 RAD52-GFP::HIS3M × 6*	This study
CCY118	*MAT***a** *ADE2 cdc21::natMX leu2::LEU2-GAL-hENT1 LYS2 RAD5 trp1::TRP1-GAL-dNK ura3-1 sgs1∆kanMX*	This study
CCY193	*MAT***a** *ADE2 cdc21::natMX leu2::LEU2-GAL-hENT1 LYS2 RAD5 trp1::TRP1-GAL-dNK ura3-1 rad52∆kanMX*	This study
CCY150	*MAT***a** *ADE2 cdc21::kanMX leu2::LEU2-GAL-hENT1 LYS2 RAD5 trp1::TRP1-GAL-dNK ura3-1 rad51∆natMX*	This study
CCY182	*MAT***a** *ADE2 cdc21::kanMX leu2::LEU2-GAL-hENT1 LYS2 RAD5 trp1::TRP1-GAL-dNK ura3-1 rad52-Y66A*	This study
MCY736	*MAT***a**/*MAT*α *ade2-1*/*ade2-1 can1-100*/*can1-100 his3-11,15*/*his3-11,15 leu2-3,112*/*leu2-3,112 trp1-1*/*trp1-1 ura3-1*/*ura3-1 RAD5*/*RAD5 mus81ΔkanMX*/*MUS81 rad52∆*/*RAD52 sgs1ΔHIS3*/*SGS1*	This study
MCY737	*MAT***a**/*MAT*α *ade2-1*/*ade2-1 can1-100*/*can1-100 his3-11,15*/*his3-11,15 leu2-3,112*/*leu2-3,112 trp1-1*/*trp1-1 ura3-1*/*ura3-1 RAD5*/*RAD5 mus81ΔkanMX*/*MUS81 rad51∆natMX*/*RAD51 sgs1ΔHIS3*/*SGS1*	This study
MCY773	*MAT***a**/*MAT*α *ade2-1*/*ade2-1 can1-100*/*can1-100 his3-11,15*/*his3-11,15 leu2-3,112*/*leu2-3,112 trp1-1*/*trp1-1 ura3-1*/*ura3-1 RAD5*/*RAD5 rad52-Y66A::hphMX*/*RAD52 sgs1ΔHIS3*/*SGS1*	This study
CCY198	*MAT***a**/*MAT*α *ade2-1*/*ade2-1 can1-100*/*can1-100 his3-11,15*/*his3-11,15 leu2-3,112*/*leu2-3,112 trp1-1*/*trp1-1 ura3-1*/*ura3-1 RAD5*/*RAD5 rad52-Y66A*/*RAD52 rmi1ΔkanMX*/*RMI1*	This study
MCY735	*MAT***a**/*MAT*α *ade2-1*/*ade2-1 can1-100*/*can1-100 his3-11,15*/*his3-11,15 leu2-3,112*/*leu2-3,112 trp1-1*/*trp1-1 ura3-1*/*ura3-1 RAD5*/*RAD5 mus81ΔkanMX*/*MUS81 rad52-R70A*/*RAD52 sgs1ΔHIS3*/*SGS1*	This study
CCY196	*MAT***a**/*MAT*α *ade2-1*/*ade2-1 can1-100*/*can1-100 his3-11,15*/*his3-11,15 leu2-3,112*/*leu2-3,112 trp1-1*/*trp1-1 ura3-1*/*ura3-1 RAD5*/*RAD5 rad52-R70A*/*RAD52 rmi1ΔkanMX*/*RMI1*	This study

### Isolation of single cells for Strand-seq

**Figure 6.**
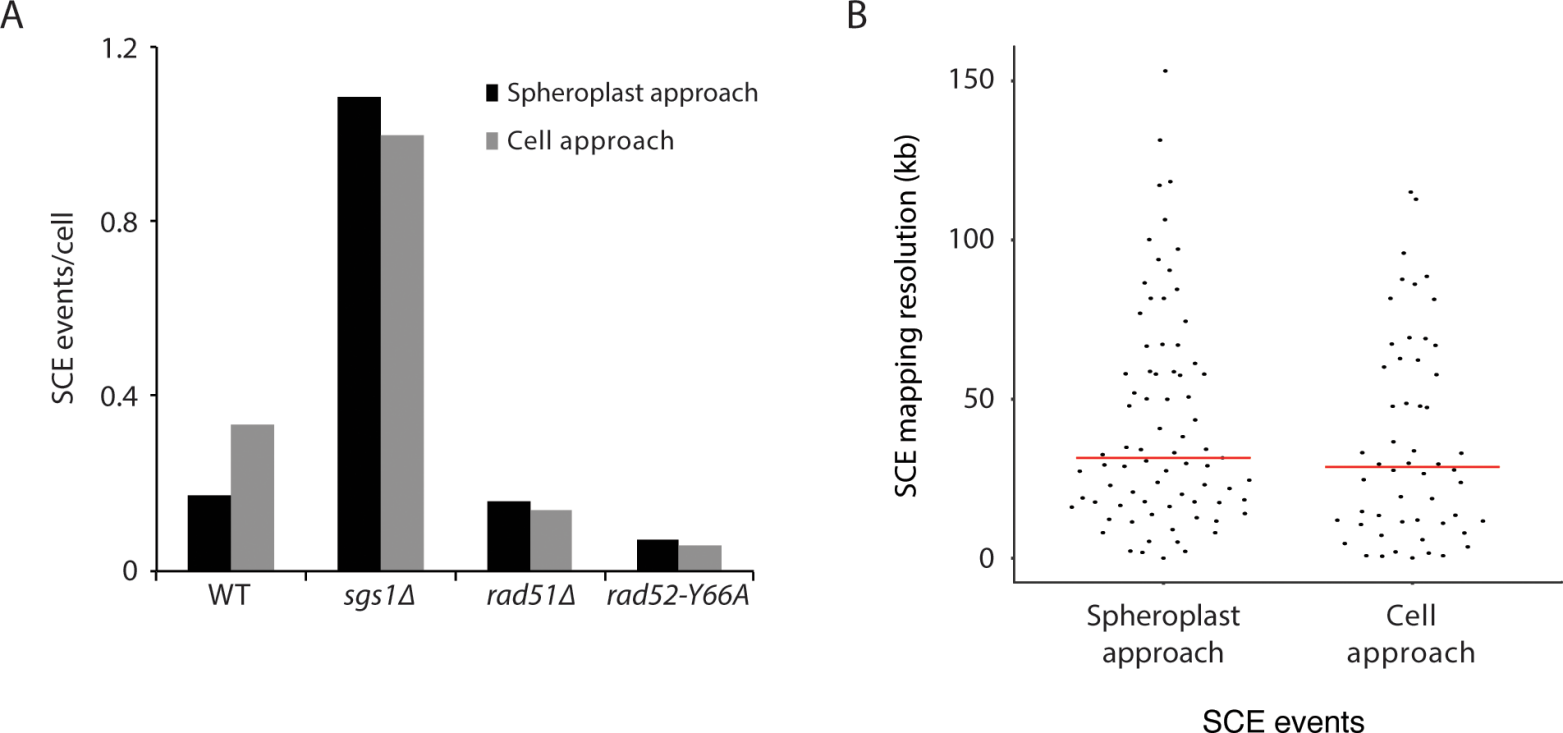
The spheroplast-sorting approach compared to the cell-sorting approach in terms of (**A**) number of SCE events per cell (divided by genotype) and (**B**) SCE mapping resolution.

Strand-seq compatible strains (*cdc21::kanMX leu2::LEU2-GAL-hENT1 trp1::TRP1-GAL-dNK*) were grown in 25 ml of YP medium (1% yeast extract, 2% peptone) supplemented with 2% galactose (YPGal) and 100 µM thymidine to an OD_600_ of 0.2–0.4. Cells were arrested in G1 phase by the addition of alpha mating factor (Sigma) for 2 hr. At this point, two different protocols were used: the first involved sorting single cells, while the second involved sorting individual spheroplasts. Both protocols yielded high quality Strand-seq libraries, with no change in the number of SCE events observed per cell (Figure 6A), nor in SCE mapping resolution (Figure 6B), but the spheroplast approach proved to be more reliable with less background sequencing reads. The Strand-seq library shown in Figure 1B was derived from the cell sorting approach, while the Strand-seq libraries shown in Figure 1C and D were derived from the spheroplast sorting approach.

**Figure 7.**
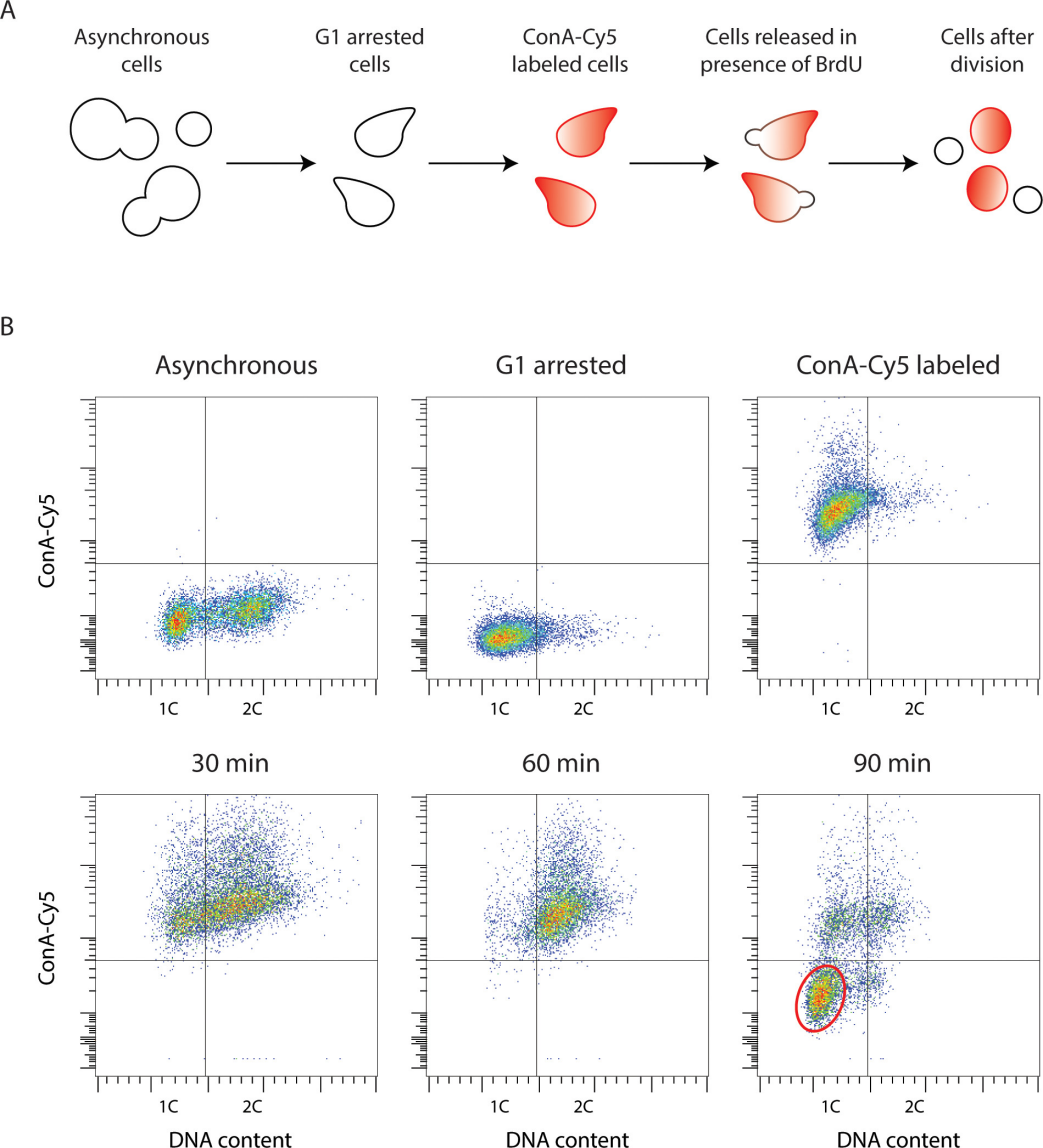
Yeast Strand-seq via the 'single cell sorting' approach. (**A**) Scheme of the ‘single cell sorting’ approach for yeast Strand-seq. (**B**) Representative flow cytometry scatter plots corresponding to the scheme depicted in (**A**). Cells appearing in the lower left quadrant in the 90 min post-release sample are newly divided daughter cells to be sorted. A typical window for sorting is shown by the red oval.

For the cell sorting approach, G1-arrested cells were washed with water and resuspended in 100 µl of PBS solution. 150 µl of 200 µg/ml of Concanavalin A (ConA), conjugated to either Cy5 or Alexa Fluor 633, was then added, followed by a 10 min incubation at room temperature in the dark. ConA binds to the yeast cell wall (Tkacz et al., 1971). Unbound ConA was removed by washing three times with PBS. Cells were then synchronously released from G1 arrest into fresh YPGal supplemented with 0.6 mg/ml of BrdU. Samples were taken before G1 arrest, after G1 arrest, after ConA-labeling, and every 30 min after release from G1 arrest. The samples were fixed with 70% ethanol and stored for at least one night at −20°C. Cells were pelleted, resuspended in 0.5 ml of 50 mM Tris-Cl pH 8.0 plus 10 µl of 10 mg/ml RNase A, and incubated for 2–4 hr at 37°C. The cells were then washed once with 50 mM Tris-Cl pH 7.5, resuspended in 300 µl of 50 mM Tris-Cl pH 7.5, 1.5 µM SYTOX Green, and incubated overnight at 4°C. Cell suspensions were briefly sonicated and analyzed by flow cytometry. Newly divided daughter cells can be identified by having 1C DNA content and without ConA labeling, since yeast daughter cells bud off from the original mother cells, synthesizing a new cell wall that is not stained with ConA (Chung et al., 1965; Figure 7). The single daughter cells were then sorted with a MoFlo Astrios cell sorter (Beckman Coulter) into separate wells of a 96-well plate for Strand-seq library preparation.

For the spheroplast sorting approach, G1 arrest and release into BrdU-containing media was performed as in the cell sorting protocol, with the omission of ConA-labeling. Cell cycle progression was monitored by flow cytometry. Cells that had progressed through one cell cycle were incubated 0.17 mg/ml zymolyase for 1 hr at 37°C. The resulting spheroplasts were washed in PBS and those with 1C DNA content were sorted with a FACSJazz cell sorter (BD Biosciences) into separate wells of a 96-well plate for Strand-seq library preparation. Without the ConA-labeling, we cannot distinguish newly divided cells from cells that remain arrested in G1 without having progressed through the cell cycle. However, the arrested cells will not have incorporated BrdU, resulting in Strand-seq libraries that will have both Watson and Crick reads for every chromosome. These libraries can be easily identified and discarded. Moreover, we find that the occurrence of such libraries is very rare.

### Strand-seq library preparation and sequencing

Strand-seq libraries were prepared as previously described (Sanders et al., 2017), with a few modifications. Sorted single yeast cells were first treated with zymolyase, followed by incubation with MNase to fragment the DNA. Several zymolyase reaction conditions were used in an attempt to optimize the procedure. In the end, we found that 0.175 mg/ml zymolyase for 30 min at 30°C worked best. Sorted single spheroplasts were directly subjected to MNase treatment. Adapter ligation was performed in the presence of PEG 6000, using the Quick Ligation Reaction Buffer (New England Biolabs) and 10 nM adapters to promote efficient ligation of adapters to DNA templates and reduce adapter dimer formation. After ligation and PCR, DNA clean-up with magnetic AMPure XP beads (Agencourt AMPure, Beckman Coulter) was performed twice using a 1.2 vol of beads. Single-end 50 bp sequencing reads from the Strand-seq libraries were generated using the HiSeq 2500 or the NextSeq 500 sequencing platform (Illumina, San Diego, CA). Reads were aligned to the *S. cerevisiae* EF4 reference genome using Bowtie2 (version 2.0.0-beta7; Langmead and Salzberg, 2012) and analyzed using the BAIT software package (Hills et al., 2013) for data selection and visualization purposes. Only libraries with an average read count greater than 20 per megabase of DNA were included in the analyses. SCE events were detected using an R-based package called HapSCElocatoR (publicly available through GitHub: https://github.com/daewoooo/HapSCElocatoR; Porubský and Sanders, 2017 ; copy archived at https://github.com/elifesciences-publications/HapSCElocatoR). HapSCElocatoR uses circular binary segmentation (implemented in the R package fastseg; Klambauer et al., 2012) to localize SCE events in haploid Strand-seq libraries as a change in read directionality from Watson (negative strand) to Crick (positive strand) or vice versa. Only non-duplicate reads with a mapping quality greater than or equal to 10 were analyzed. We considered only strand state changes with at least three directional reads on both sides of the putative SCE site as an SCE event. Single directional reads embedded within an extended region with the opposite directionality were considered as errors and their directionality was flipped. Computationally localized SCE events were further manually verified by visual inspection of chromosome ideograms (obtained from BAIT; see Figure 1B). Comparisons of SCE rate between different genotypes were assessed for statistical significance using a *t*-test.

### Live cell imaging of cells expressing Rad52-GFP

Strains CCY232 and CCY234 were inoculated in the morning in 2 mL of synthetic medium supplemented with 100 µM thymidine and grown at 25°C. In the evening, the cultures were diluted into 50 mL of synthetic medium supplemented with thymidine to reach an OD_600_ of 0.2–0.4 in the following morning. Cells were then washed twice with synthetic medium and placed either into 25 mL of synthetic medium supplemented with 100 µM thymidine (for CCY232 and CCY234) or 25 mL of synthetic medium supplemented with 200 µM BrdU (for CCY232) and grown for 90 min at 25°C—instead of 30°C, to allow the GFP chromophore to fold more efficiently (Lim et al., 1995). Cells were then mounted onto agar pads for imaging, which was performed using a Zeiss LSM 800 or a DeltaVision Image Restoration Microscope. Images were scores for cells with or without foci by two people independently.

### Estimation of spontaneous DSB rate

Freshly grown E17 (wild type) or CCY193 (*rad52∆*) were spread onto one side of a YPGal +100 µM thymidine plate. Cells were micromanipulated to designated locations on the plate and allowed to divide once. If a cell failed to divide at this stage, it was discarded from further analysis. This step ensured that we would look at healthy and newly divided cells. From the cells that do divide, each daughter cell was then micromanipulated away from its mother cell to a new location on the same plate, which was then incubated for 2–3 days at 30°C to allow the isolated mother and daughter cells to form colonies. The number of mother or daughter cells that were unable to form a colony was recorded. Incidences where both the mother and the daughter did not form a colony were excluded from the analysis because these mother-daughter pairs may have resulted from the division of an arrested cell with unrepaired DNA damage as a result of DNA damage checkpoint adaptation (Clémenson and Marsolier-Kergoat, 2009). In total, 408 E17 and 338 CCY193 cells were examined (i.e. 204 E17 and 169 CCY193 mother-daughter pairs). 13.3% (95% CI [9.9%, 17.4%]) of CCY193 cells and 1.7% (95% CI [0.7%, 3.5%]) of E17 cells failed to form a colony. E17 mortality is unlikely due to unrepaired DSBs, and possibly due to death caused by the micromanipulation process, so subtracting this 1.7% ‘background’ mortality rate from the 13.3% mortality rate of CCY193 cells yields an estimated spontaneous DSB rate of 11.6%. This mortality rate is similar to the 8.3% previously reported for *rad52∆* of a different strain background (Ozenberger and Roeder, 1991).

## Additional files

Transparent reporting form

## References

[bib1] Bai Y, Symington LS (1996). A Rad52 homolog is required for *RAD51*-independent mitotic recombination in *Saccharomyces cerevisiae*. Genes & Development.

[bib2] Chaganti RS, Schonberg S, German J (1974). A manyfold increase in sister chromatid exchanges in Bloom's syndrome lymphocytes. PNAS.

[bib3] Chang M, Bellaoui M, Zhang C, Desai R, Morozov P, Delgado-Cruzata L, Rothstein R, Freyer GA, Boone C, Brown GW (2005). *RMI1/NCE4*, a suppressor of genome instability, encodes a member of the RecQ helicase/Topo III complex. The EMBO Journal.

[bib4] Chung KL, Hawirko RZ, Isaac PK (1965). Cell wall replication in *Saccharomyces cerevisiae*. Canadian Journal of Microbiology.

[bib5] Clémenson C, Marsolier-Kergoat MC (2009). DNA damage checkpoint inactivation: adaptation and recovery. DNA Repair.

[bib6] Coïc E, Feldman T, Landman AS, Haber JE (2008). Mechanisms of Rad52-independent spontaneous and UV-induced mitotic recombination in *Saccharomyces cerevisiae*. Genetics.

[bib7] Davis AP, Symington LS (2001). The yeast recombinational repair protein Rad59 interacts with Rad52 and stimulates single-strand annealing. Genetics.

[bib8] Donnianni RA, Symington LS (2013). Break-induced replication occurs by conservative DNA synthesis. PNAS.

[bib9] Fabre F, Chan A, Heyer WD, Gangloff S (2002). Alternate pathways involving Sgs1/Top3, Mus81/ Mms4, and Srs2 prevent formation of toxic recombination intermediates from single-stranded gaps created by DNA replication. PNAS.

[bib10] Falconer E, Hills M, Naumann U, Poon SS, Chavez EA, Sanders AD, Zhao Y, Hirst M, Lansdorp PM (2012). DNA template strand sequencing of single-cells maps genomic rearrangements at high resolution. Nature Methods.

[bib11] Fasullo M, Giallanza P, Dong Z, Cera C, Bennett T (2001). *Saccharomyces cerevisiae rad51* mutants are defective in DNA damage-associated sister chromatid exchanges but exhibit increased rates of homology-directed translocations. Genetics.

[bib12] Fasullo MT, Davis RW (1987). Recombinational substrates designed to study recombination between unique and repetitive sequences *in vivo*. PNAS.

[bib13] Fasullo MT, Sun M (2017). Both *RAD5*-dependent and independent pathways are involved in DNA damage-associated sister chromatid exchange in budding yeast. AIMS genetics.

[bib14] Gangavarapu V, Prakash S, Prakash L (2007). Requirement of *RAD52* group genes for postreplication repair of UV-damaged DNA in *Saccharomyces cerevisiae*. Molecular and Cellular Biology.

[bib15] Gangloff S, McDonald JP, Bendixen C, Arthur L, Rothstein R (1994). The yeast type I topoisomerase Top3 interacts with Sgs1, a DNA helicase homolog: a potential eukaryotic reverse gyrase. Molecular and Cellular Biology.

[bib16] German J, Archibald R, Bloom D (1965). Chromosomal breakage in a rare and probably genetically determined syndrome of man. Science.

[bib17] González-Barrera S, Cortés-Ledesma F, Wellinger RE, Aguilera A (2003). Equal sister chromatid exchange is a major mechanism of double-strand break repair in yeast. Molecular Cell.

[bib18] Hills M, O'Neill K, Falconer E, Brinkman R, Lansdorp PM (2013). BAIT: Organizing genomes and mapping rearrangements in single cells. Genome Medicine.

[bib19] Holliday R (1964). A mechanism for gene conversion in fungi. Genetical Research.

[bib20] Ira G, Malkova A, Liberi G, Foiani M, Haber JE (2003). Srs2 and Sgs1-Top3 suppress crossovers during double-strand break repair in yeast. Cell.

[bib21] Jackson JA, Fink GR (1981). Gene conversion between duplicated genetic elements in yeast. Nature.

[bib22] Jasin M, Rothstein R (2013). Repair of strand breaks by homologous recombination. Cold Spring Harbor Perspectives in Biology.

[bib23] Johnson RD, Jasin M (2000). Sister chromatid gene conversion is a prominent double-strand break repair pathway in mammalian cells. The EMBO Journal.

[bib24] Kadyk LC, Hartwell LH (1992). Sister chromatids are preferred over homologs as substrates for recombinational repair in *Saccharomyces cerevisiae*. Genetics.

[bib25] Klambauer G, Schwarzbauer K, Mayr A, Clevert DA, Mitterecker A, Bodenhofer U, Hochreiter S (2012). cn.MOPS: mixture of Poissons for discovering copy number variations in next-generation sequencing data with a low false discovery rate. Nucleic Acids Research.

[bib26] Kobayashi T (2011). Regulation of ribosomal RNA gene copy number and its role in modulating genome integrity and evolutionary adaptability in yeast. Cellular and Molecular Life Sciences.

[bib27] Kong F, Zhu J, Wu J, Peng J, Wang Y, Wang Q, Fu S, Yuan LL, Li T (2011). dbCRID: a database of chromosomal rearrangements in human diseases. Nucleic Acids Research.

[bib28] Labib K, Hodgson B (2007). Replication fork barriers: pausing for a break or stalling for time?. EMBO reports.

[bib29] Lambert S, Mizuno K, Blaisonneau J, Martineau S, Chanet R, Fréon K, Murray JM, Carr AM, Baldacci G (2010). Homologous recombination restarts blocked replication forks at the expense of genome rearrangements by template exchange. Molecular Cell.

[bib30] Langmead B, Salzberg SL (2012). Fast gapped-read alignment with Bowtie 2. Nature Methods.

[bib31] Lettier G, Feng Q, de Mayolo AA, Erdeniz N, Reid RJ, Lisby M, Mortensen UH, Rothstein R (2006). The role of DNA double-strand breaks in spontaneous homologous recombination in *S. cerevisiae*. PLoS Genetics.

[bib32] Lim CR, Kimata Y, Oka M, Nomaguchi K, Kohno K (1995). Thermosensitivity of green fluorescent protein fluorescence utilized to reveal novel nuclear-like compartments in a mutant nucleoporin NSP1. The Journal of Biochemistry.

[bib33] Lisby M, Rothstein R, Mortensen UH (2001). Rad52 forms DNA repair and recombination centers during S phase. PNAS.

[bib34] Mazina OM, Keskin H, Hanamshet K, Storici F, Mazin AV (2017). Rad52 inverse strand exchange drives RNA-Templated DNA double-strand break repair. Molecular Cell.

[bib35] Mehta A, Haber JE (2014). Sources of DNA double-strand breaks and models of recombinational DNA repair. Cold Spring Harbor Perspectives in Biology.

[bib36] Meselson MS, Radding CM (1975). A general model for genetic recombination. PNAS.

[bib37] Mortensen UH, Bendixen C, Sunjevaric I, Rothstein R (1996). DNA strand annealing is promoted by the yeast Rad52 protein. PNAS.

[bib38] Mortensen UH, Erdeniz N, Feng Q, Rothstein R (2002). A molecular genetic dissection of the evolutionarily conserved N terminus of yeast Rad52. Genetics.

[bib39] Mott C, Symington LS (2011). *RAD51*-independent inverted-repeat recombination by a strand-annealing mechanism. DNA Repair.

[bib40] Muñoz-Galván S, Jimeno S, Rothstein R, Aguilera A (2013). Histone H3K56 acetylation, Rad52, and non-DNA repair factors control double-strand break repair choice with the sister chromatid. PLoS Genetics.

[bib41] Mullen JR, Nallaseth FS, Lan YQ, Slagle CE, Brill SJ (2005). Yeast Rmi1/Nce4 controls genome stability as a subunit of the Sgs1-Top3 complex. Molecular and Cellular Biology.

[bib42] Nassif N, Penney J, Pal S, Engels WR, Gloor GB (1994). Efficient copying of nonhomologous sequences from ectopic sites via P-element-induced gap repair. Molecular and Cellular Biology.

[bib43] New JH, Sugiyama T, Zaitseva E, Kowalczykowski SC (1998). Rad52 protein stimulates DNA strand exchange by Rad51 and replication protein A. Nature.

[bib44] Onoda F, Seki M, Miyajima A, Enomoto T (2000). Elevation of sister chromatid exchange in *Saccharomyces cerevisiae sgs1* disruptants and the relevance of the disruptants as a system to evaluate mutations in Bloom's syndrome gene. Mutation Research/DNA Repair.

[bib45] Ozenberger BA, Roeder GS (1991). A unique pathway of double-strand break repair operates in tandemly repeated genes. Molecular and Cellular Biology.

[bib46] Persky NS, Lovett ST (2008). Mechanisms of recombination: lessons from *E. coli*. Critical Reviews in Biochemistry and Molecular Biology.

[bib47] Petukhova G, Stratton SA, Sung P (1999). Single strand DNA binding and annealing activities in the yeast recombination factor Rad59. Journal of Biological Chemistry.

[bib48] Porubský D, Sanders AD (2017). HapSCElocatoR. GitHub.

[bib49] Prakash R, Zhang Y, Feng W, Jasin M (2015). Homologous recombination and human health: the roles of BRCA1, BRCA2, and associated proteins. Cold Spring Harbor Perspectives in Biology.

[bib50] Radding CM (1982). Homologous pairing and strand exchange in genetic recombination. Annual Review of Genetics.

[bib51] Rattray AJ, Symington LS (1994). Use of a chromosomal inverted repeat to demonstrate that the *RAD51* and *RAD52* genes of *Saccharomyces cerevisiae* have different roles in mitotic recombination. Genetics.

[bib52] Saini N, Ramakrishnan S, Elango R, Ayyar S, Zhang Y, Deem A, Ira G, Haber JE, Lobachev KS, Malkova A (2013). Migrating bubble during break-induced replication drives conservative DNA synthesis. Nature.

[bib53] Sanders AD, Falconer E, Hills M, Spierings DCJ, Lansdorp PM (2017). Single-cell template strand sequencing by Strand-seq enables the characterization of individual homologs. Nature Protocols.

[bib54] Sasaki M, Kobayashi T (2017). Ctf4 Prevents Genome Rearrangements by Suppressing DNA Double-Strand Break Formation and Its End Resection at Arrested Replication Forks. Molecular Cell.

[bib55] Sherman F (2002). Getting started with yeast. Methods in Enzymology.

[bib56] Shi I, Hallwyl SC, Seong C, Mortensen U, Rothstein R, Sung P (2009). Role of the Rad52 amino-terminal DNA binding activity in DNA strand capture in homologous recombination. Journal of Biological Chemistry.

[bib57] Shinohara A, Shinohara M, Ohta T, Matsuda S, Ogawa T (1998). Rad52 forms ring structures and co-operates with RPA in single-strand DNA annealing. Genes to Cells.

[bib58] Shor E, Gangloff S, Wagner M, Weinstein J, Price G, Rothstein R (2002). Mutations in homologous recombination genes rescue *top3* slow growth in *Saccharomyces cerevisiae*. Genetics.

[bib59] Stark JM, Jasin M (2003). Extensive loss of heterozygosity is suppressed during homologous repair of chromosomal breaks. Molecular and Cellular Biology.

[bib60] Sugiyama T, New JH, Kowalczykowski SC (1998). DNA annealing by RAD52 protein is stimulated by specific interaction with the complex of replication protein A and single-stranded DNA. PNAS.

[bib61] Sung P (1997). Function of yeast Rad52 protein as a mediator between replication protein A and the Rad51 recombinase. Journal of Biological Chemistry.

[bib62] Symington LS, Rothstein R, Lisby M (2014). Mechanisms and regulation of mitotic recombination in *Saccharomyces cerevisiae*. Genetics.

[bib63] Thomas BJ, Rothstein R (1989). Elevated recombination rates in transcriptionally active DNA. Cell.

[bib64] Tkacz JS, Cybulska EB, Lampen JO (1971). Specific staining of wall mannan in yeast cells with fluorescein-conjugated concanavalin A. Journal of Bacteriology.

[bib65] van Wietmarschen N, Lansdorp PM (2016). Bromodeoxyuridine does not contribute to sister chromatid exchange events in normal or Bloom syndrome cells. Nucleic Acids Research.

[bib66] Vernis L (2003). Reconstitution of an efficient thymidine salvage pathway in *Saccharomyces cerevisiae*. Nucleic Acids Research.

[bib67] Virgin JB, Bailey JP, Hasteh F, Neville J, Cole A, Tromp G (2001). Crossing over is rarely associated with mitotic intragenic recombination in *Schizosaccharomyces pombe*. Genetics.

[bib68] Weiffenbach B, Haber JE (1981). Homothallic mating type switching generates lethal chromosome breaks in *rad52* strains of *Saccharomyces cerevisiae*. Molecular and Cellular Biology.

[bib69] Wu L, Hickson ID (2003). The Bloom's syndrome helicase suppresses crossing over during homologous recombination. Nature.

[bib70] Wu Y, Sugiyama T, Kowalczykowski SC (2006). DNA annealing mediated by Rad52 and Rad59 proteins. Journal of Biological Chemistry.

[bib71] Xu X, Blackwell S, Lin A, Li F, Qin Z, Xiao W (2015). Error-free DNA-damage tolerance in *Saccharomyces cerevisiae*. Mutation Research/Reviews in Mutation Research.

[bib72] Zhao X, Muller EG, Rothstein R (1998). A suppressor of two essential checkpoint genes identifies a novel protein that negatively affects dNTP pools. Molecular Cell.

[bib73] Zou H, Rothstein R (1997). Holliday junctions accumulate in replication mutants via a RecA homolog-independent mechanism. Cell.

